# Sexual Functions Following Cardiac Resynchronization Therapy: Evaluation of Male Patients and Their Partners

**DOI:** 10.7759/cureus.40163

**Published:** 2023-06-08

**Authors:** Halil F Oncel, Remzi Salar, Ömer F Cicek

**Affiliations:** 1 Urology, Mehmet Akif İnan Resarch and Training Hospital, Sanliurfa, TUR; 2 Urology, Mehmet Akif İnan Training and Research Hospital, Sanliurfa, TUR; 3 Cardiology, Mehmet Akif İnan Resarch and Training Hospital, Sanliurfa, TUR

**Keywords:** male sexual health, female sexual function, cardiac resynchronization, erectile dysfunction, heart failure

## Abstract

Introduction: Sexual functionality, a critical component of health-related quality of life, can decline for various reasons, including heart failure (HF). Our purpose was to prospectively evaluate male patients with HF scheduled for cardiac resynchronization therapy (CRT) in terms of sexual function, erectile function, and alterations in hormonal and biochemical parameters. In addition, we sought to determine the sexual functioning of the partners of these patients.

Methods: The study enrolled 103 male patients and their partners. The International Index of Erectile Function-5 (IIEF-5) was administered to all males and the Arizona Sexual Experience Scale (ASEX) questionnaire was completed by all participants, at baseline and three months after CRT.

Results: The ASEX scores of patients and partners demonstrated significant declines from baseline to post-intervention analysis. The IIEF-5 scores of patients showed a significant increase from baseline to post-intervention (p=0.001 for all).

Conclusion: We conclude that sexual dysfunction is experienced by the partners of male patients with erectile dysfunction before CRT and that reversal of erectile problems with CRT yields improvements in both male and female sexual functions.

## Introduction

The inability to achieve or sustain a penile erection that allows satisfactory sexual functioning is defined as erectile dysfunction (ED) when it occurs perpetually [1]. Although ED is estimated to be relatively common, patients rarely report their complaints directly to their physician(s). Therefore, physicians need to identify patients’ status by asking appropriate questions [1]. To achieve an erection, penile structures (vascular and neural) must function normally, and thus, problems in the function of these structures are recognized as factors associated with ED [2]. ED is seen in one in two men older than 40 years [3]. Age, cardiovascular diseases and accompanying drugs, i.e. b-blockers, diabetes, hypercholesterolemia, smoking, depression and psychiatric diseases, psychological disorders, and adverse socioeconomic conditions are risk factors for ED [4].

Heart failure (HF) is characterized by its multifactorial origin and effects on different systems; however, the triggering mechanism is usually a result of cardiovascular diseases, such as long-lasting arterial hypertension and myocardial infarction. Although HF is classified according to various features, the primary form of HF (left ventricular) is associated with decreased myocardial contractility which leads to reduced cardiac output, increased end-diastolic volume, and diminished cardiac reserve [5]. In HF, sexual dysfunction can manifest as ED or difficulties in performance and orgasm [6]. Healthy sexual functioning is dependent upon psychological, neurological, vascular, and hormonal factors, which must operate in unison to achieve and sustain an erection, but the primary trigger is the flow of arterial blood into the corpora cavernosa and spongiosum [7]. The disruption of this mechanism may lead to vasculogenic ED. Previous studies have associated ED with cardiovascular diseases, and it is recommended that patients with ED be screened for cardiac diseases [8].

Vasculogenic ED, which occurs due to vascular problems (inflow or outflow), is now considered to be responsible for the majority of cases with organic ED [9]. Atherosclerotic disease-related events (e.g., ischemic stroke, myocardial infarction) and cardiovascular risk factors have been associated with vasculogenic ED [8,10]. Additionally, predisposing factors are similar in patients with HF and ED; however, ED may also occur as a direct result of HF-related characteristics, including loss of functional capacity, medications used for HF, and the biochemical basis of the disease [11,12]. Studies have determined that CRT improves endothelial function by creating vasodilation through increased blood flow. Abrahams et al. reported that although the CRT mode was turned off, many patients showed improvement in the symptoms of HF [13,14]. It has been demonstrated that CRT improves cardiac function in around 70% of patients with HF, and increases overall physical capacity and fitness over time [15]. For instance, different studies have shown that CRT leads to better outcomes among subjects with HF, including better functional capacity, oxygen consumption during exercise, six-minute walking distance, and quality of life [16,17].

HF, often described as the “malignant” chronic disease of cardiology, causes a restriction in the filling or pumping functions of the heart and adversely affects the functional status and life span of the patient [18,19]. HF can impose considerable adverse effects on lifestyle and daily functions, including an increased likelihood of ED, which can decrease quality of life (QoL) and may lead to depression in patients. Having HF can also lead to other critical adversities in sexual functioning, including reduced sexual intercourse frequency, decreased libido, poor sexual performance, and loss of satisfaction from sexual functions [20]. Biventricular pacing, also known as cardiac resynchronization therapy (CRT), has been recognized as one of the most important developments within the last decade in the treatment of HF with wide QRS complexes and decreased ejection fraction (EF) [14]. Approximately 70% of patients with HF are recognized to experience ED, and the presence of ED is established as a contributor to worse QoL in patients with HF [21]. Our aim of this study was to evaluate changes in the sexual functions of male patients with HF who were treated by CRT and to also determine the effects of these changes on the sexual functions of their partners.

## Materials and methods

This prospectively designed research was carried out as a joint project by the Urology and Cardiology Departments of Sanliurfa Mehmet Akif İnan Training and Research Hospital (affiliated with Health Sciences University). Ethical approval was obtained from the Ethics Committee of Harran Faculty of Medicine (approval number: HRU/22/06/12). All steps of the research conformed to the Declaration of Helsinki, and written informed consent was obtained from all participants. A total of 103 male patients (and their partners) who were scheduled for CRT between January and May of 2022 at two cardiology centers affiliated with our hospital were included in the study. Patients with the following characteristics were excluded from the study: having a history of severe psychiatric disease, pelvic trauma or penile-testicular cancer, having undergone genitourinary surgery, receiving exogenous hormones (steroids, testosterone, etc.), phosphodiesterase type 5 inhibitors, and current smoking. For the partners, the exclusion criteria were having a history of severe psychiatric disease, ovarian-uterine cancer, or pelvic trauma, and having received radiotherapy to the genitourinary area.

The Arizona Sexual Experience Scale (ASEX) and the International Index of Erectile Function-5 (IIEF-5) questionnaire were applied to all patients at baseline and three months after CRT. The partners of patients completed the ASEX at the same time points.

The ASEX is a validated scale used to measure sexual dysfunction [22]. The validity and reliability of ASEX in the Turkish language have been demonstrated by Soykan [23]. Normally, when evaluating ASEX results, the presence of three items with a score of ≥5 or ≥4 for any item or a total score of ≥11 defines sexual dysfunction; however, none of the participants reported such a result. Therefore, in patients and partners, the presence of a total ASEX score of ≥11 was defined as the threshold for identifying sexual dysfunction. The IIEF-5 questionnaire is considered to be a diagnostic measure for ED, and the Turkish version of the IIEF-5 was used in this study, as validated by Turunç et al. [24]. Higher scores indicate better erectile function. Results were categorized as severe (5-7 points), moderate (8-11), mild-moderate (12-16), mild (17-21), and no complaints (22-25).

After 12 hours of fasting, venous blood samples were obtained between 7 and 11 am, placed in tubes, and centrifuged at 1500 rpm for 10 minutes. Sera were immediately separated and stored at -80°C. Testosterone, T3, T4, and thyroid-stimulating hormone (TSH) values ​​were measured with an ARCHITECT i2000SR analyzer (Abbott Diagnostics) using the immunoassay method. Lipids and glucose were measured with the colorimetric method using the ARCHITECT C16000 device (Abbott Diagnostics).

Statistical analysis

The IBM SPSS version 22.0 software (IBM Corp, Armonk, NY) was used and all analyses were subject to a significance threshold of <0.05 (p-value). Continuous variables found to have a normal distribution (Kolmogorov-Smirnov) were analyzed with parametric tests, while those without normal distribution were analyzed with non-parametric tests. Descriptive statistical methods were employed to depict data with mean and standard deviation values. The paired-sample t-test was used for the comparison of pre-treatment and post-treatment values of variables with a normal distribution, while the Wilcoxon signed-ranks test was used for non-normally distributed parameters.

## Results

A total of 103 cases aged 40-60 years who had undergone CRT between January and May 2022, and their partners, were included in the study. The demographic characteristics of the patients are shown in Table 1.

**Table 1:** Demographic Characteristics of the Patients BMI: body mass index; HF: heart failure; DM: diabetes mellitus; COPD: chronic obstructive pulmonary disease; NYHA: New York Heart Association; ACE-ARB: angiotensin-converting enzyme inhibitors - angiotensin receptor blockers.

	N=103, (%)
Age (Years) (Mean ± SD)	53.24 ± 5.75
BMI kg/m^2 ^(Mean ± SD)	28.21 ± 3.72
HF Time (Months) (Mean ± SD)	17 ± 5.75
NYHA Class	
II	27 (26.2%)
II-III	16 (15.5%)
III	51 (49.5%)
Ambulatory IV	9 (8.7%)
Additional Diseases	
DM	32 (31.1%)
Hypertension	64 (62.1%)
COPD	26 (25.2%)
Medication	
Beta-Blocker	94 (91.2%)
ACE-ARB	87 (84.4%)
Digoxin	56 (54.3%)
Spironolactone	33 (32%)
Furosemide	82 (79.6%)
Statin	54 (52.4%)
Thiazide	23 (22.3%)
Aspirin	78 (75.7%)

The mean age of the cases was 53.24 ± 5.75 years. As a result of the power analysis, the number of patients for an effect size of g = 0.25 (large effect size), power 1-b = 0.80, and a = 0.05 was determined as 101 (Table 2).

**Table 2:** Post-treatment Changes in IIEF and ASEX Scores Compared to Pre-treatment Scores Mann-Whitney U test was used; **p < 0.01. SD: standard deviation; IIEF: International Index of Erectile Function; ASEX: Arizona Sexual Experience Scale; ED: erectile dysfunction.

		Pre-treatment	Post-treatment	p-Value
		Mean ± SD (Median)	Mean ± SD (Median)	
IIEF		7.43 ± 3.77 (6)	21.84 ± 6.08 (24)	0.001**
ASEX (Patient)		18.05 ± 5.63 (11)	9.09 ± 2.7 (5)	0.001**
ASEX (Partner)		18.68 ± 7.28 (9)	10.85 ± 3.99 (7)	0.001**
		n; %	n; %	
IIEF	Severe ED	80; 77.7	5; 4.9	
	Moderate ED	19; 18.4	18; 17.5	
	Mild-Moderate ED	4; 3.9	9; 8.7	
	Mild ED	-	39; 37.9	
	No ED	-	32; 31.1	
ASEX (Patient)	<11	-	-	
	≥11	77; 74.8	26; 25.2	
ASEX (Partner)	<11	2; 1.9	66; 64.1	
	≥11	101; 98.1	37; 35.9	

**Figure 1:**
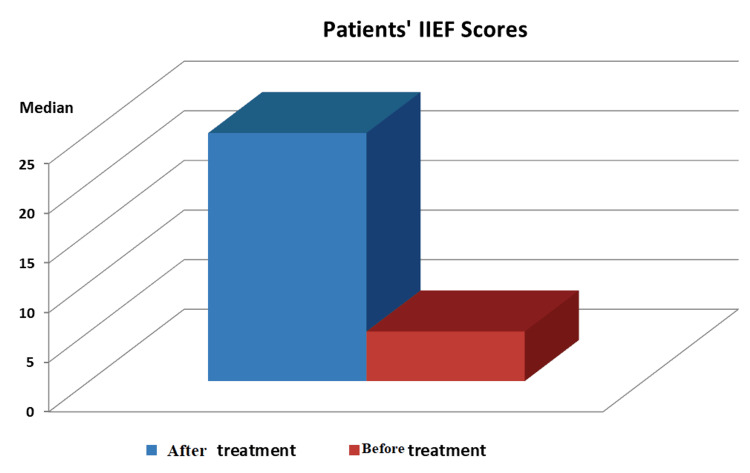
Distribution of IIEF Scores IIEF: International Index of Erectile Function.

While the mean IIEF score in the patients was 7.43 ± 3.77 before treatment, with a median of 6, it increased to 21.84 ± 6.08 (median: 24) after treatment (p < 0.01) (Figure 1).

**Figure 2:**
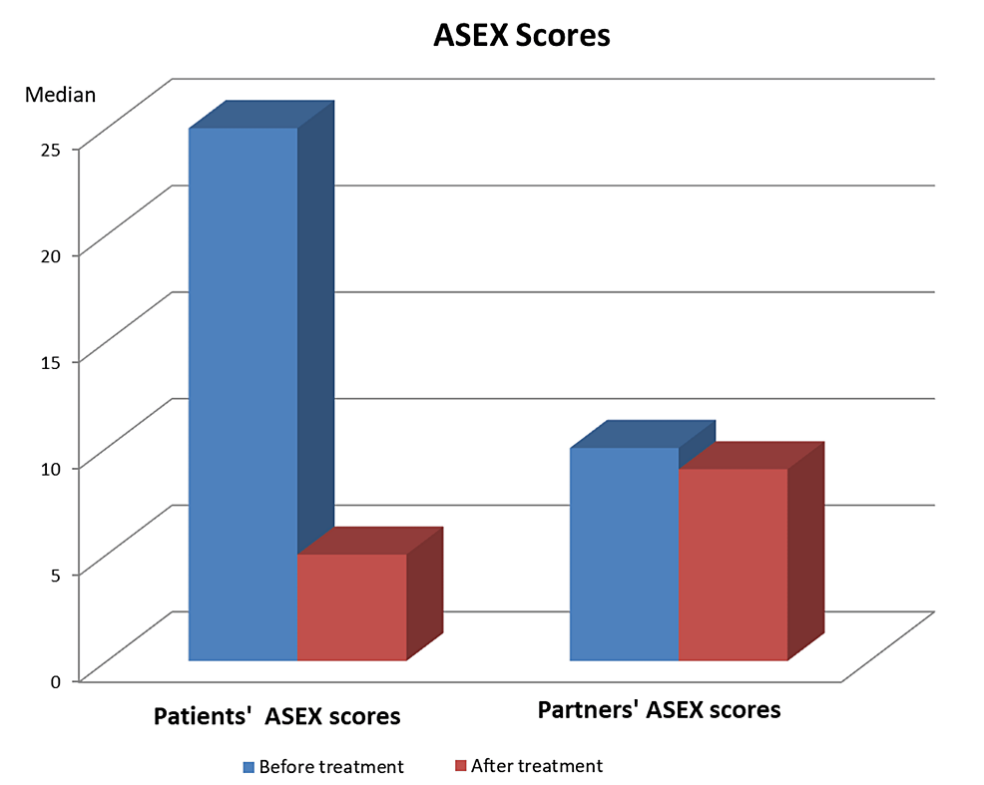
Distribution of ASEX Scores ASEX: Arizona Sexual Experience Scale.

The mean pre-treatment ASEX score of the patients was 18.05 ± 5.63 (median: 11) before treatment, which decreased to 9.09 ± 2.7 (median: 5) after treatment (p < 0.01) (Table 2). Among the partners, the mean pre-treatment and post-treatment ASEX scores were 18.68 ± 7.28 (median: 9) and 10.85 ± 3.99 (median: 7), indicating a significant decrease (Figure 2).

Significant decreases were also observed in the triglyceride, cholesterol, and low-density lipoprotein levels of the patients after treatment compared to pre-treatment values (p < 0.01). In addition, compared to pre-treatment measurements, there was a significant increase in the high-density lipoprotein (HDL) levels of the patients after treatment (p < 0.05) (Table 3).

**Table 3:** Post-treatment Changes in Blood Values Compared to Pre-treatment Evaluation ^1^Mann-Whitney U-test; ^2^Paired-samples t-test; **p < 0.01; *p < 0.05. SD: standard deviation' LDL: low-density lipoprotein; HDL: high-density lipoprotein; TSH: thyroid-stimulating hormone.

	Pre-treatment	Post-treatment	p-Value
	Mean ± SD (Median)	Mean ± SD (Median)	
Testosterone	4.07 ± 0.9 (3.9)	4.18 ± 0.92 (4.2)	^1^0.146
Triglyceride	200.06 ± 24.24 (201)	185.5 ± 19.62 (186)	^1^0.001**
Cholesterol	206.46 ± 21.9 (198)	189.38 ± 21.11 (190)	^1^0.001**
LDL	134.35 ± 18.31 (132)	130.02 ± 16.02 (128)	^1^0.001**
HDL	43.73 ± 6.47 (44)	44.94 ± 7.79 (48)	^1^0.010*
TSH	3.35 ± 6.27 (2.1)	3.15 ± 5.49 (2)	^1^0.004**
T3	6.92 ± 7.39 (4.9)	5.25 ± 3.58 (4.8)	^1^0.001**
T4	16.72 ± 1.49 (16.3)	17.48 ± 1.34 (17.9)	^1^0.001**
Glucose	103.12 ± 18.37	103.38 ± 5.29	^2^0.877

## Discussion

We found that CRT performed due to HF improved the sexual functions of male patients. In addition, unlike similar studies, we evaluated changes in the sexual functions of the partners of CRT recipients for the first time in the literature. We also found improved post-intervention sexual functions among the partners of male patients who had undergone CRT.

In a study evaluating the sexual functions of HF patients, Jaarsma et al. determined a linear relationship between EF and sexual function. In addition, the authors noted that subjects with HF had decreased libido, reduced coitus frequency and sexual satisfaction, and adverse changes in sexual function [25]. ED may not always be the first symptom of patients, but when the cause/underlying cause of ED is corrected, a significant improvement can also be seen in their primary complaints. In a study evaluating bariatric surgery recipients and their partners, the sexual satisfaction of both groups was found to have increased after surgery [26]. In another study, it was found that the IIEF-5 scores significantly improved after the correction of thyroidism in patients with dysthyroidism presenting with ED [27]. Studies have also shown considerable benefits with the utilization of HF treatment before starting patients on ED treatment [28]. Kuyumcu et al. reported that patients responsive to CRT had significant post-treatment improvements in the scores obtained from the Sexual Health Inventory for Men questionnaire [11]. In a similar study by Vural et al., left ventricular EF and ED improvement were found to demonstrate a significant correlation [7]. Baumhäkel et al., who evaluated ED patients with high cardiovascular risk, divided patients into three groups according to ED severity and ventricular function. The authors reported a significant increase in ED risk among patients with moderate or severe left ventricular dysfunction. Furthermore, ED development was found to be independently associated with left ventricular dysfunction [29]. In our study, we found that ED symptoms improved with the correction of HR, which also resulted in increased sexual satisfaction for both the patients and their partners.

ASEX is used to evaluate the sexual functions/satisfaction of male and female patients [23]. ASEX questions various parameters to determine various elements of sexual function, including libido, arousal, penile erection, vaginal lubrication, orgasm, and satisfaction in both men and women [30]. In studies evaluating patients with HF, sexual problems affecting QoL were reported by 52% of males and 38% of females, as well as worsening of psychological status and relationship satisfaction [31-33]. A study by Baert et al. reported that almost half of the HF patients attributed worsening sexual activity to their disease, while more than a third considered that HF was responsible for a significant decrease in sexual pleasure, loss of interest, and persistent problems related to engaging in sexual activity [34]. In the evaluation of sexual anxiety among patients with HF, the partner perspective should also be given importance and taken into account by healthcare providers [6]. As such, it is evident that positive changes in the sexual health of patients will also have a positive effect on the sexual satisfaction of their partners. In our study, in parallel with the improvements in the ASEX scores of male patients after CRT, we also observed positive alterations in the ASEX scores of partners.

The treatment of chronic diseases, such as diabetes, hypertension, hyperlipidemia, hypothyroidism, depression, and low testosterone, can improve ED symptoms [1]. In a prospective study investigating changes in the laboratory parameters of 129 patients (mostly men) after CRT, Boros et al. reported a lipid profile similar to our results. However, the authors did not examine sex hormone levels [35]. The present analyses did not reveal a significant change in testosterone levels before and after CRT treatment, but we found a significant decrease in patients’ triglyceride, LDH, and total cholesterol levels and a significant increase in their HDL levels. In addition, we detected a significant decrease in the T3 and TSH levels, which were within the reference range, as well as a significant increase in the T4 levels. We consider that these changes also played a role in the improvement in the ED of the patients. It is well established that chronic HF is not just a loss of ventricular pump function, and therefore, it results in various systemic effects. Additionally, through the activation of the immune system, it can also cause metabolic disorders that can be monitored through various laboratory parameters [36,37].

A primary limitation of our study is the lack of evaluation with color Doppler ultrasonography to assess penile structures. However, this was largely due to the invasive nature of the procedure. In addition, we did not evaluate whether the psychological states of the patients and their partners were affected by the procedure.

## Conclusions

CRT improves ED and increases sexual satisfaction in male patients by increasing cardiac output and affecting the lipid profile. This also indirectly leads to an increase in the sexual satisfaction of partners. There is a need for prospectively designed placebo-controlled double-blind randomized studies with larger case series.
